# Proline oxidase controls proline, glutamate, and glutamine cellular concentrations in a U87 glioblastoma cell line

**DOI:** 10.1371/journal.pone.0196283

**Published:** 2018-04-25

**Authors:** Pamela Cappelletti, Elena Tallarita, Valentina Rabattoni, Paola Campomenosi, Silvia Sacchi, Loredano Pollegioni

**Affiliations:** 1 Department of Biotechnology and Life Sciences, University of Insubria, Varese, Italy; 2 The Protein Factory Research Center, Politecnico of Milano and University of Insubria, Milano, Italy; Rutgers University, UNITED STATES

## Abstract

L-Proline is a multifunctional amino acid that plays an essential role in primary metabolism and physiological functions. Proline is oxidized to glutamate in the mitochondria and the FAD-containing enzyme proline oxidase (PO) catalyzes the first step in L-proline degradation pathway. Alterations in proline metabolism have been described in various human diseases, such as hyperprolinemia type I, velo-cardio-facial syndrome/Di George syndrome, schizophrenia and cancer. In particular, the mutation giving rise to the substitution *Leu441Pro* was identified in patients suffering of schizophrenia and hyperprolinemia type I. Here, we report on the expression of wild-type and *L441P* variants of human PO in a U87 glioblastoma human cell line in an attempt to assess their effect on glutamate metabolism. The subcellular localization of the flavoenzyme is not altered in the *L441P* variant, for which specific activity is halved compared to the wild-type PO. While this decrease in activity is significantly less than that previously proposed, an effect of the substitution on the enzyme stability is also apparent in our studies. At 24 hours of growth from transient transfection, the intracellular level of proline, glutamate, and glutamine is decreased in cells expressing the PO variants as compared to control U87 cells, reaching a similar figure at 72 h. On the other hand, the extracellular levels of the three selected amino acids show a similar time course for all clones. Furthermore, PO overexpression does not modify to a significant extent the expression of GLAST and GLT-1 glutamate transporters. Altogether, these results demonstrate that the proline pathway links cellular proline levels with those of glutamate and glutamine. On this side, PO might play a regulatory role in glutamatergic neurotransmission by affecting the cellular concentration of glutamate.

## Introduction

**Fig 1:**
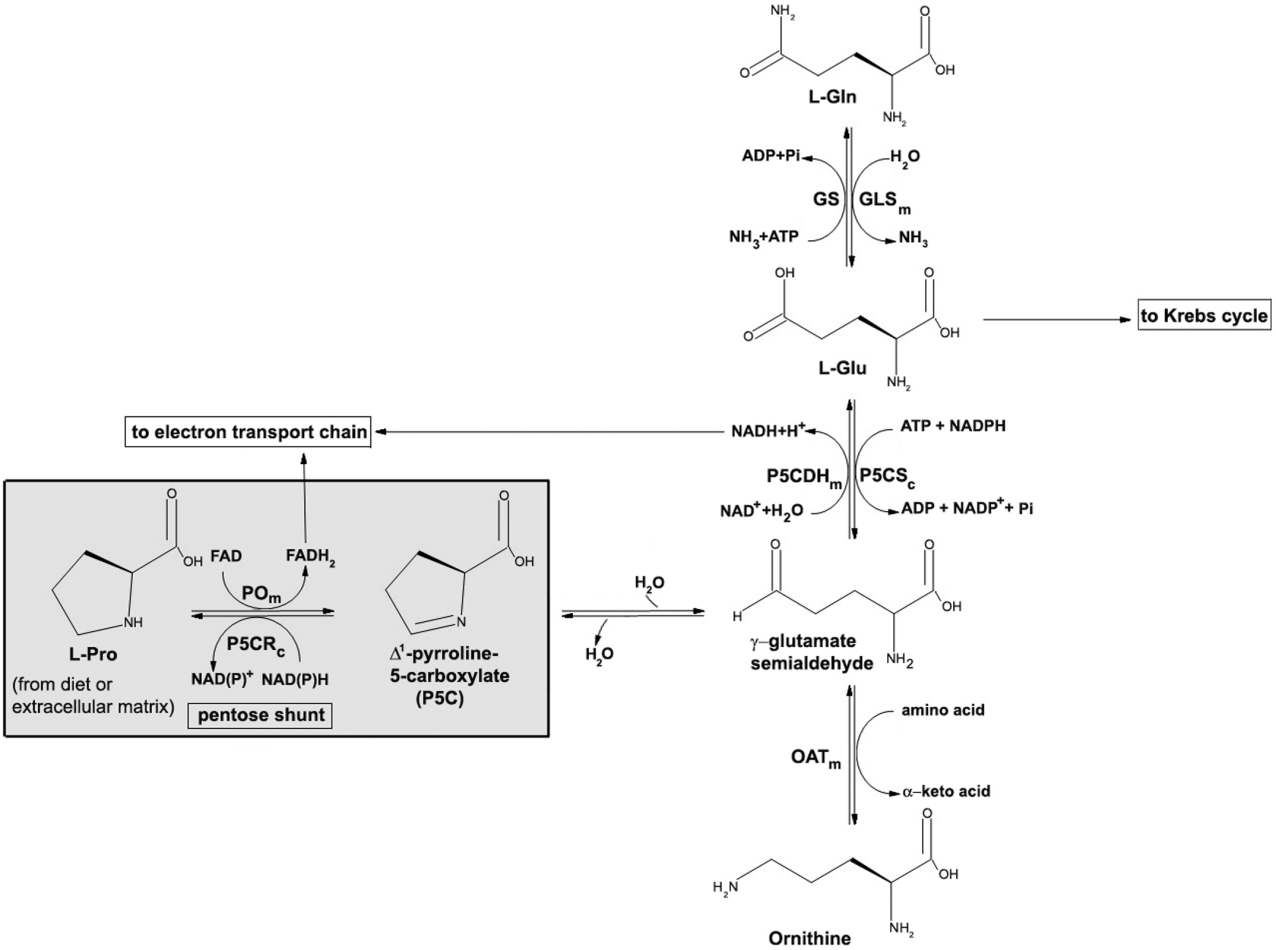
The proline metabolic pathway and its crosslinks with other metabolic pathways. PO: proline oxidase; P5CR: pyrroline-5-carboxylate reductase 1; P5CDH: Δ^1^-pyrroline-5-carboxylate dehydrogenase; P5CS: pyrroline-5-carboxylate synthase; GS: glutamine synthase; GLS: glutaminase; OAT: ornithine aminotransferase. The subfix m indicates mitochondrial localization; the subfix c indicates cytosolic localization. The step catalyzed by PO is highlighted by a grey box.

Proline is a nonessential amino acid with a distinctive cyclic structure. It plays a central role in metabolism and is increasingly being recognized as a critical amino acid in physiology, such as bioenergetics, cellular redox control, and apoptosis, as well as in pathology [1–3]. Owing to its unique chemical structure (it is an imino acid), proline metabolism is distinct from that of proteinogenic amino acids: intracellular synthesis and degradation occur through a distinct set of enzymes with unique properties and regulatory mechanisms [1, 4]. Proline can be endogenously synthesized either from glutamate or ornithine, and it is also readily available from the breakdown of the extracellular matrix (Fig 1). The first step of degradation, which takes place in the inner mitochondrial membrane, is performed by the FAD-dependent enzyme proline oxidase (PO, EC 1.5.99.8), also known as proline dehydrogenase (PRODH). It catalyzes the oxidation of L-proline to Δ^1^-pyrroline-5-carboxylate (P5C), a key metabolite with several metabolic destinations (Fig 1): i) nonenzymatic hydrolysis to glutamate semialdehyde, that can be further oxidized to the neurotransmitter glutamate by mitochondrial P5C dehydrogenase (P5CDH, EC 1.5.1.12); ii) converted to ornithine by ornithine aminotransferase (OAT, EC 2.6.1.13); iii) reduced back to proline by cytosolic P5C reductase (P5CR, EC 1.5.1.2) [3, 5–7]. Noteworthy, glutamate and glutamine are mutually converted by the reaction catalyzed by glutamine synthetase (GS, EC 6.3.1.2) and glutaminase (GLS, EC 3.5.1.2), see Fig 1.

In addition to be linked to the metabolism of the neurotransmitter glutamate, a role of proline in neurotransmission has been proposed: high proline concentrations affect glutamate release [8] and have a neurotoxic effect [9]. In addition, a high-affinity transporter for proline has been identified, supporting a role of proline in brain function [10–12].

The *PRODH* gene, encoding PO, is widely expressed in brain and other tissues and is a “hotspot” for mutations. At least 16 missense mutations, most of which reach polymorphic frequencies, have been identified in individuals suffering from hyperprolinemia type I (HPI, MIM 239500) or schizophrenia [13–16]. Indeed, five of the identified mutations, among which the one resulting in the *Leu441Pro* (*L441P*) substitution, were proposed to negatively affect the flavoenzyme activity (reduction > 70%) [17]. HPI-affected individuals show a tenfold increase in proline concentration in plasma and cerebral spinal fluid [18] and some cognitive disfunction [1, 19].

In order to investigate the structure-function relationships in PO, we previously produced in *E*. *coli* and characterized a recombinant human PO deletion variant, namely, PO-barrelN-His, encompassing the whole catalytic domain [20]. In the present study, we report on the expression of the full-length wild-type PO and its *L441P* variant in the U87 glioblastoma human cell line. We investigated the expression levels and subcellular localization of the two PO variants in clones stably expressing the flavoprotein, even in the chimeric form fused to the enhanced yellow fluorescent protein (EYFP). Furthermore, we analyzed the intra- and extracellular concentrations of proline, glutamine, and glutamate as related to overexpression of the PO variants. Our results highlight the importance of PO in determining cellular levels of proline, thus suggesting that the enzyme might play a regulatory role in the complex processes related to glutamatergic neurotransmission.

## Materials and methods

### Expression vector

The design for cDNA coding the full-length human PO was based on the sequence information reported in [16]. The data bank of Source Bioscience—I.M.A.G.E. (Integrated Molecular Analysis of Genomes and their Expression) reported different cDNAs corresponding to *PRODH* gene. By aligning the I.M.A.G.E. *PRODH* sequence, two clones with partially overlapping regions (pBlue–IRAKp961I0147Q and pCR-BluntII-TOPO-IRCMp5012A1133D) were identified, containing the 5’- and the 3’-terminus portions of the expected full-length *PRODH* sequence (the one encoding for human PO, 600 amino acids). The DNA from these two clones was digested with EcoRI and BbvCI restriction enzymes (Thermo Scientific). The fragments of interest were subsequently ligated (T4 DNA ligase, Roche) into pGEMT-easy and the sequence of the final construct was confirmed by automated sequencing. The resulting DNA encoding human wild-type PO was amplified by PCR (using the following primers: 5’–cataaagcttatggctctgaggcgcgccctg-3’; 5’-gttggtaccaaggcagggcgatggaagaggttg-3’) and subcloned into HindIII and KpnI restriction sites of the pEYFP-N1 expression vector (Clontech), allowing the upstream expression of PO fused to EYFP. In order to produce the untagged flavoprotein, the PO encoding DNA was subcloned from pGEMT-easy into the pcDNA3 vector (employing the EcoRI restriction sites). The *L441P* mutation was introduced into the pEYFP-N1-prodh or pcDNA3-prodh constructs by using the QuikChange Site-Directed Mutagenesis Kit (Stratagene); successful mutagenesis was confirmed by DNA sequencing.

### Cell culture and transfection

The U87 human glioblastoma cells (ATCC) were maintained in DMEM supplemented with 10% fetal bovine serum (FBS), 2 mM L-glutamine, 1% penicillin/streptomycin, and 1% amphotericin B (Euroclone) at 37 °C in a 5% CO_2_ incubator. U87 cells were transfected using the FuGene^®^HD transfection reagent (Promega, 8 μL) and 2 μg of the following constructs: pEYFP-N1-prodh, pEYFP-N1-prodh-*L441P*, pcDNA3-prodh, pcDNA3-prodh-*L441P*, and pcDNA3 (empty). Transfection efficiency of U87 cells in the aforementioned conditions was determined by using an EYFP expressing construct: transfected cells were analyzed with a transmitted light fluorescence microscope and the fraction of fluorescent cells over total cells in five different fields of view was scored. Transfection efficiency was determined to be in the 30–40% range. Cell clones stably expressing the different protein variants (namely, PO-EYFP, PO-EYFP *L441P*, PO, and PO *L441P*) were selected by adding 0.4 mg/mL G418 (Euroclone) to the growth medium. Protein expression level was monitored either by a fluorescence microscope (Olimpus IX51) to detect the EYFP associated signal or by Western blot analysis (see below). The expression of the PO variants in the selected cell clones was checked after thawing and before starting each experiment. The levels of PO variants expression appeared stable after multiple subculture passages.

### Cell growth curves

Stocks of cell clones stably transfected with pcDNA3, pcDNA3-prodh, or pcDNA3-prodh-*L441P* preserved in liquid nitrogen were thawed, splitted once, then seeded in 96-well plates (1.5 x 10^3^ cells/well) and incubated at 37 °C in a 5% CO2 incubator. Cell growth was monitored for 12 days (with no further splitting and medium replacement) and measured every 24 hours as follows: the growth medium of the selected well was replaced with a solution containing 0.5 mg/mL MTT (3-[4,5-dimethylthiazol-2-yl]-2,5-diphenyltetrazolium bromide) prepared in PBS, and the plates were incubated in the dark for 4 hours at 37 °C. The solution was then discarded and the formed formazan crystals were solubilized using 100 μL dimethylsulfoxide: formazan concentration was measured by recording the changes in absorbance at 600 nm using a microplate reader (Infinite M200, Tecan). Each clone was analyzed in triplicate at each time point. Growth curve data were expressed as absorbance/well vs. time.

### Western blot analysis

Stably/transiently transfected cells grown as reported above were detached by trypsin treatment, counted, harvested, and resuspended in Laemmli 2x sample buffer. An amount of sample corresponding to 5 x 10^4^ or 1 x 10^5^ cells of each clone was separated by SDS-PAGE electrophoresis and transferred to a polyvinylidene difluoride membrane (Immobilon-P, Millipore). Western blot analyses were carried out, after blocking the membranes overnight at 4 °C with 4% dried milk or 5% bovine serum albumin diluted in phosphate buffer saline (PBS: 10 mM sodium phosphate dibasic, 1.75 mM potassium phosphate monobasic, 137 mM NaCl, and 2.7 mM KCl, pH 7.4) to which 0.1% Tween-20 was added (TPBS), or in Tris-buffered saline (TBS: 10 mM Tris-HCl, pH 8.0, 0.5 M NaCl) to which 0.1% Tween-20 was added (TTBS). Subsequently, the membranes were incubated with primary antibodies for 1.5 h at room temperature or 37 °C, washed extensively with TPBS or TTBS, and incubated for 1 h at room temperature with specific peroxidase–conjugated secondary antibodies. After washing, the immunoreactivity signals were detected by enhanced chemiluminescence (ECL plus, GE Healthcare). The immunoreactivity signals were detected using the following primary antibodies: rabbit polyclonal anti-PO (1:1000, Davids Biotecnologie); mouse monoclonal anti-GFP (1:500, SantaCruz); rabbit polyclonal anti-actin (1:1000, Sigma); rabbit polyclonal anti-GLT1 (anti-EAAT2, 1:1200, Abnova); rabbit polyclonal anti-GLAST (anti-EAAT1, 1:1000, Thermo Scientific); and mouse monoclonal anti-ERAB (1:100, SantaCruz). The immunorecognition signals were quantified using the Quantity One software (BioRad): the intensity values were normalized to the values obtained for the anti-actin antibody.

### Determination of cellular and extracellular concentration of selected amino acids by HPLC analysis

U87 cells were seeded in 6-well plates (2.5 x 10^5^ cells/well) and transiently transfected with pcDNA3, pcDNA3-prodh, or pcDNA3-prodh-*L441P* constructs: the medium has been never replaced during the experiment. At 24, 48, and 72 hours after transfection, both the culture medium and the cells were collected and used for HPLC analysis of the amino acids content. The culture medium was diluted with HCl (25 mM final concentration) and centrifuged at 39000 *g* for 30 min at 4 °C, while the cells were detached by trypsin treatment (2 min at room temperature), harvested, washed with ice cold PBS and rapidly frozen at -80 °C before further analysis. This procedure of cell recovery was preferred to mechanical treatment because resulted in a higher yield of amino acid recovery, see S1 Fig and S1 Table. Cell extracts were prepared as reported previously [21]: the cells were resuspended (2.5 x 10^5^ cells/mL) in ice-cold 5% trichloroacetic acid, sonicated (three cycles of 30 s each, on ice, using a Branson Sonifier 250; Branson Ultrasonics, Danbury, CT, USA), and centrifuged for 30 min at 16000 *g*. The soluble fraction was extracted with water-saturated ether (1:1) and lyophilized. Samples were derivatized using the solution included in the Waters AccQ Tag^™^ Amino Acid Analysis kit, following the manufacturer’s instructions: this procedure converts both primary and secondary amino acids in stable derivatives (fluorescence at 395 nm following excitation at 250 nm). Briefly, samples were diluted in “AccQ-Fluor Borate buffer”, vortexed and added of 20 μL of “AccQ-Fluor Reagent”, vortexed, incubated 1 min at room temperature and then 10 min at 55 °C. Derivatized samples were resolved by HPLC chromatography on a 4-μm C18 reverse-phase column (3.9 x 150 mm, Waters) and eluted by a gradient (from 100% aqueous “Waters AccQ Tag^™^ buffer” to 60% acetonitrile: 40% milliQ water in 36 minutes) at 37 °C. Each amino acid was identified and quantified according to the retention time and peak area. The calibration curves were obtained by injecting increasing concentrations of each amino acid.

### Immunofluorescence

For immunofluorescence, 2.5 x 10^5^ U87 cells stably expressing PO-EYFP or PO-EYFP *L441P* were seeded on round, glass coverslips and incubated for 24 hours at 37 °C and 5% CO_2_. The cells were then extensively washed with PBS solution (10 mM sodium phosphate dibasic, 2 mM potassium phosphate monobasic, 137 mM NaCl, and 2.7 mM KCl, pH 7.4) and fixed with 4% *p*-formaldehyde and 4% sucrose in 0.1% sodium phosphate buffer, pH 7.4, for 15 min at room temperature. Fixed cells were permeabilized and the unspecific binding sites were blocked by a 30-min incubation in PBS supplemented with 4% horse serum and 0.2% Triton X-100. The coverslips were incubated for 24 or 48 h at 4 °C with primary antibodies raised against a specific mitochondrial marker (1:50 anti-mitochondria antibody, surface intact mitochondria, clone 113–1, Millipore), or the glutamate transporters GLAST (anti-EAAT1 1:200, Thermo Scientific) and GLT-1 (anti-EAAT2 1:20, Abnova). Immunoreactivity was detected with donkey anti-mouse Alexa 546-conjugated antibody (1:1000, Invitrogen) and donkey anti-rabbit Alexa 647-conjugated antibody (1:1000, Invitrogen). Immunostained coverslips were imaged using an inverted laser scanning confocal microscope (TCS SP5, Leica Microsystems equipped with a 63.0 x 1.25 NA plan apochromate oil immersion objective). Confocal image stacks were collected with Leica SP5 software, avoiding interference between each channel and without saturating any pixel.

### Mitochondria isolation

A subcellular fraction enriched in intact mitochondria was prepared from U87 cells transiently transfected with pcDNA3-prodh, pcDNA3-prodh-*L441P*, or pcDNA3 and collected 48 h after transfection by using the MACS technology (Mitochondria isolation kit, Miltenyi Biotech), which magnetically labels the organelles with anti-TOM22 antibody. Briefly, ≈ 1 x 10^7^ cells were detached by trypsin treatment, counted and washed with 10 mL ice-cold “lysis buffer” to which protease inhibitors were added (2 μM leupeptin, 1 μM pepstatin, and 500 μM phenylmethylsulfonyl fluoride); the samples were homogenized (by passing them 45 times through a 0.2-μm needle in ice). Then, 9 mL of ice-cold PBS and 50 μL of anti-TOM22 magnetic beads were added and the samples were incubated for 1 h at 4 °C before magnetically separating the mitochondria on the MACS column rinsed with “separation buffer” and then placed in the MACS separator. The column was washed three times with 3 mL of separation buffer and removed from the separator. The magnetically labeled mitochondria were flushed out by adding 1.5 mL of separation buffer. The isolated mitochondria were centrifuged at 13000 g for 2 min at 4°C, washed with 1 mL of “storage buffer”, centrifuged again at 13000 g for 2 min at 4 °C and finally suspended in 100 μL of “storage buffer” [22]. Activity assays have been carried out immediately after the mitochondrial isolation.

### PO activity assay

The enzymatic activity of PO variants expressed in U87 cell clones was measured by two methods. First, the *o*-aminobenzaldehyde (o-AB) spectrophotometric assay was used [3]. Briefly, P5C formed by enzymatic oxidation of proline was reacted with o-AB and the ensuing product was quantified, recording the absorbance at 440 nm. U87 control cells and U87 cells expressing untagged wild-type or *L441P* PO were grown in 9-cm plates up to 80% of confluence. Subsequently, cells were rinsed and scraped in cold PBS buffer, collected, and resuspended in cold sucrose buffer (250 mM sucrose, 3.5 mM Tris-HCl, and 1 mM EDTA, pH 7.4). Cells were then sonicated for 20 s and the cell extracts clarified by centrifugation at 16000 *g* for 20 min at 4 °C. A 200-μL reaction mixture containing 0.12 mg/mL o-AB, 0.012 mg/mL cytochrome c, 0.5–200 mM proline in 0.1 M phosphate buffer, pH 7.2, and a volume of cell extract corresponding to 50 μg of total proteins were incubated for 30 min at 37 °C. The reaction was blocked by adding 20 μL of o-AB (10 mg/mL in 6 N HCl). Unfortunately, despite the use of different amounts of samples (cell extract corresponding up to 50 μg of total proteins), the increase in L-Pro concentration (in the 0.5–200 mM range), and the presence of 0.5 mM exogenous FAD, we failed to obtain a consistent determination of PO activity.

The PO activity was assayed on the subcellular fraction enriched in intact mitochondria of U87 cells (see above) at 25 °C and using the oxidoreductase assay based on the decrease of the absorbance at 595 nm related to dichlorophenolindophenol (DCPIP), as previously described [20]. Briefly, L-Pro:DCPIP oxidoreductase activity assay was performed in 1 mL of reaction volume: changes in absorbance were recorded at 595 nm (ε = 16,100 M^-1^cm^-1^). The reaction mixture contained ≈ 1 μg of total mitochondrial proteins, 0.25 mM phenazine methasulfate, 50.4 μM DCPIP, 10 mM MgCl_2_, 10% glycerol, and L-Pro (100–200 mM concentration range) in 10 mM MOPS, pH 7.5, and 0.5 mM FAD [23]. One unit is defined as the amount of enzyme which transfers electrons from 1 μmol of L-Pro to DCPIP in 1 min at 25 °C. The specificity of the assay was verified by assessing the inhibition of PO activity by adding 8 mM tetrahydro-2-furoic acid [20].

All the analyses were replicated three times for each condition; variation between groups was evaluated by one-way ANOVA. The total protein content was determined using the Bradford protein assay (Sigma).

## Results

### Subcellular localization of PO variants in U87 cells

**Fig 2:**
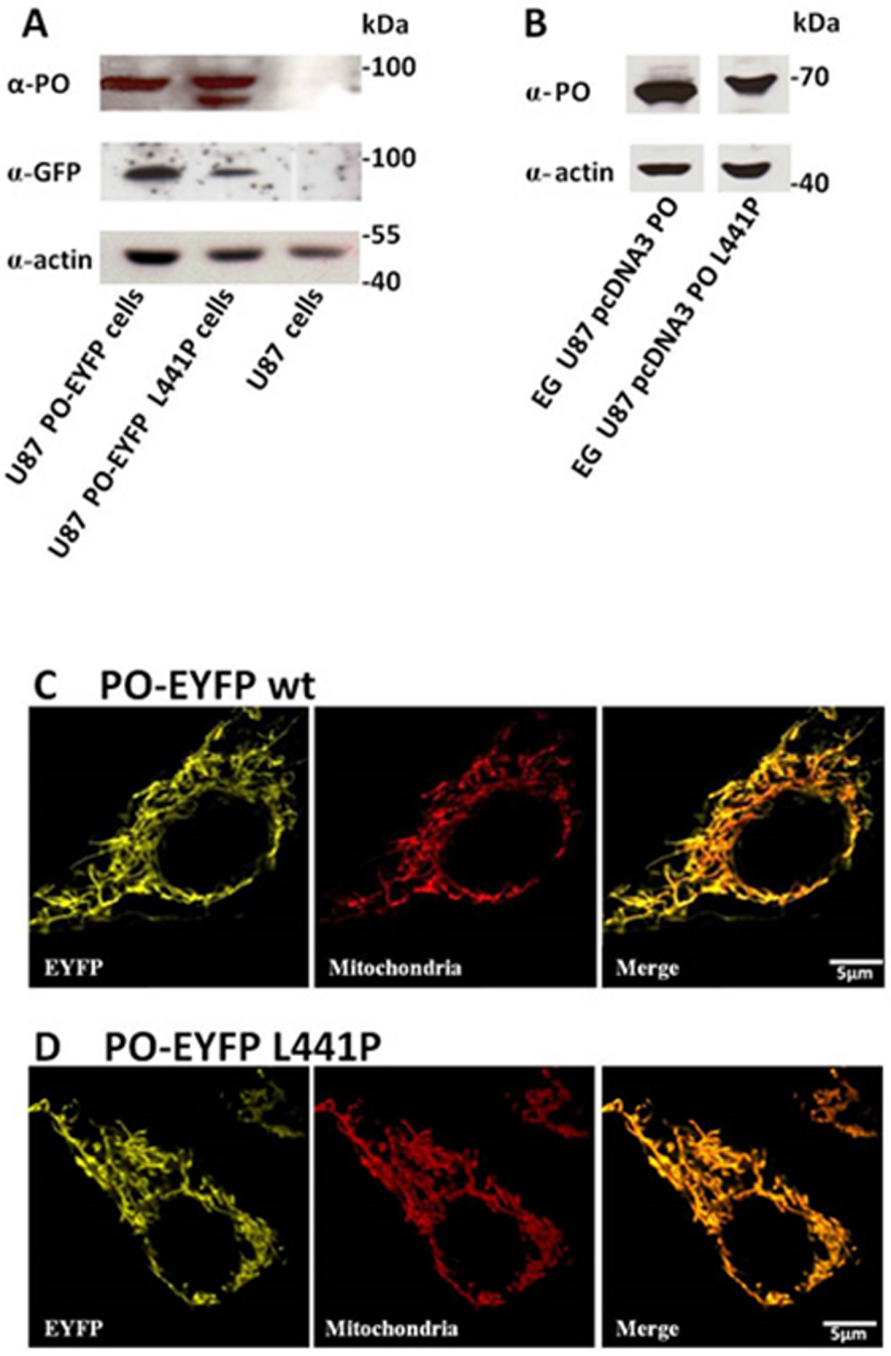
Expression of PO in stably transfected U87 clones. (A,B) Western blot analysis performed using mouse anti-GFP or rabbit anti-PO antibodies confirms the expression of both the fluorescent PO-EYFP wild-type and *L441P* fusion proteins in transfected U87 cells (A) or the untagged proteins (B). The same amount of sample, corresponding to 5 x 10^4^ cells or 50 μg of crude extract (EG), was loaded in each lane, as further confirmed by an anti-actin antibody. The level of endogenous PO in U87 control cells is below the detection limit (1 ng). The PO-EYFP *L441P* variant detected with the anti-PO antibody shows a further band at lower molecular mass corresponding to a partial proteolyzed form of the protein. (C, D) Confocal analysis of PO subcellular distribution in cultured U87 glioblastoma cells stably expressing PO-EYFP wild-type or PO-EYFP *L441P*. Both chimeric proteins display a perinuclear “spaghetti-like” distribution (yellow channel) which largely overlaps (merge panel) with the mitochondria-specific immunostaining signal (red channel). Western blot experiments were replicated four times. Scale bar: 5 μm.

The U87 human glioblastoma cell line was selected in this study to investigate PO localization and function since it represents a useful *in vitro* model of astroglia: it has been previously used to express the human flavoenzyme D-amino acid oxidase and to investigate the processes involved in the metabolism of the neuromodulator D-serine related to NMDA receptor function [21–23]. The endogenous PO expression level in control U87 cells was below detection. U87 cells were transfected with pEYFP-N1-prodh, pEYFP-N1-prodh-*L441P*, pcDNA3-prodh, or pcDNA3-prodh-*L441P* constructs encoding for EYFP tagged or untagged PO variants. Upon selection, several clones stably expressing the protein variants were obtained as confirmed by Western blot analysis: a single band corresponding to the chimeric fluorescent protein (≈ 95 kDa) was detected in U87 PO-EYFP and PO-EYFP *L441P* cells using either anti-PO or anti-GFP antibodies (Fig 2A and 2B). In U87 PO-EYFP *L441P* cell extract, the anti-PO antibody detected a further band at lower molecular mass, likely suggesting a partial degradation of the chimeric protein. Interestingly, in the same sample, the anti-GFP antibody detected only the band corresponding to the full-length chimeric fluorescent protein, indicating that the proteolytic cleavage might occur at the C-terminal end of recombinant PO and thus causing partial loss of EYFP. The observation that the expression level of EYFP-tagged wild-type and *L441P* PO are comparable when detected using the anti-PO antibody but appear lower when revealed by the anti-GFP antibody further confirms this conclusion. An anti-PO signal at the predicted molecular mass (≈ 68 kDa) was also detected in U87 PO and U87 PO *L441P* clones expressing the untagged proteins with no evidence of protein degradation (Fig 2B).

PO has been previously reported to be a mitochondrial protein [24]. As the EYFP tag might alter the subcellular targeting of overexpressed chimeric proteins, the subcellular localization of the two PO variants was verified by immunostaining experiments. Confocal analyses showed that the fluorescent signal corresponding to the chimeric proteins displays a perinuclear “spaghetti-like” distribution typical of mitochondria (Fig 2C and 2D; yellow channel). Indeed, it largely colocalized with the signal for mitochondria, stained with a specific antibody (Figs 2C and 1D; merge panel). The wild-type and the L441P PO EYFP chimeric proteins exhibited a similar subcellular localization, indicating that the protein targeting is not affected by the introduced point mutation or by the EYFP tag.

### Growth of U87 cells stably expressing wild-type or L441P PO

**Fig 3:**
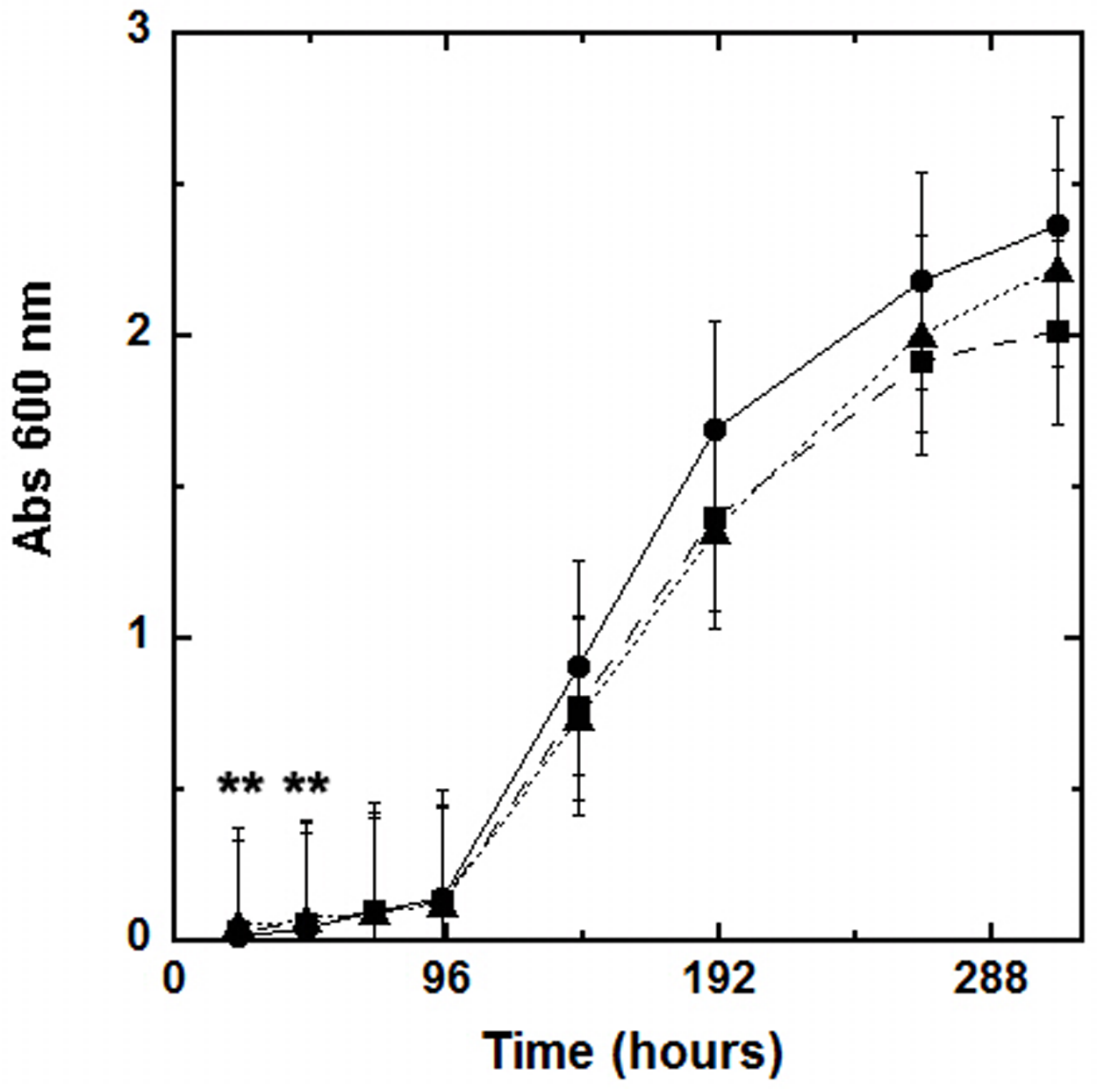
U87 cell growth curves by MTT assay. Cell growth was monitored for 12 days. Three independent U87 clones for each construct, i.e. pcDNA3 (circles, continuous line), pcDNA3-prodh-wild-type (squares, dashed line), or pCDNA3-prodh-*L441P* (triangles, dotted line), were seeded in 96-well plates (1.5 x 10^3^ cells/well) and incubated at 37 °C in a 5% CO_2_ incubator. The values of absorbance at 600 nm represent the average of three clones transfected with the same vector (mean value ± standard error, n = 6; **p ≤ 0.05 at the selected time).

We investigated the effect of the overexpression of the PO variants on the proliferation of stably transfected U87 cell clones. The U87 cells harboring the empty pcDNA3 plasmid (control) or the constructs encoding for wild-type or *L441P* PO were seeded at 10.5 x 10^3^ cells/well starting density and the cell growth rate was monitored over a period of 12 days by using the MTT method. The growth curves display an exponential trend because the nutrient sources were not depleted during the incubation period. The growth rate of U87 clones expressing the PO variants was slightly lower than that of control cells (Fig 3). The average doubling time of the PO expressing U87 cells is 1.2-fold higher with respect to U87 control cells (wild-type: 47.0 ± 10.8 hours; *L441P*: 48.0 ± 12.5 hours; control: 39.3 ± 15 hours; p ≥ 0.5, see Fig 3). Notably, the expression of the wild-type or the PO *L441P* variant has the same effect on cell growth (as indicated by the overlapping curves, Fig 3).

### Enzymatic activity in U87 cells stably expressing PO

**Fig 4:**
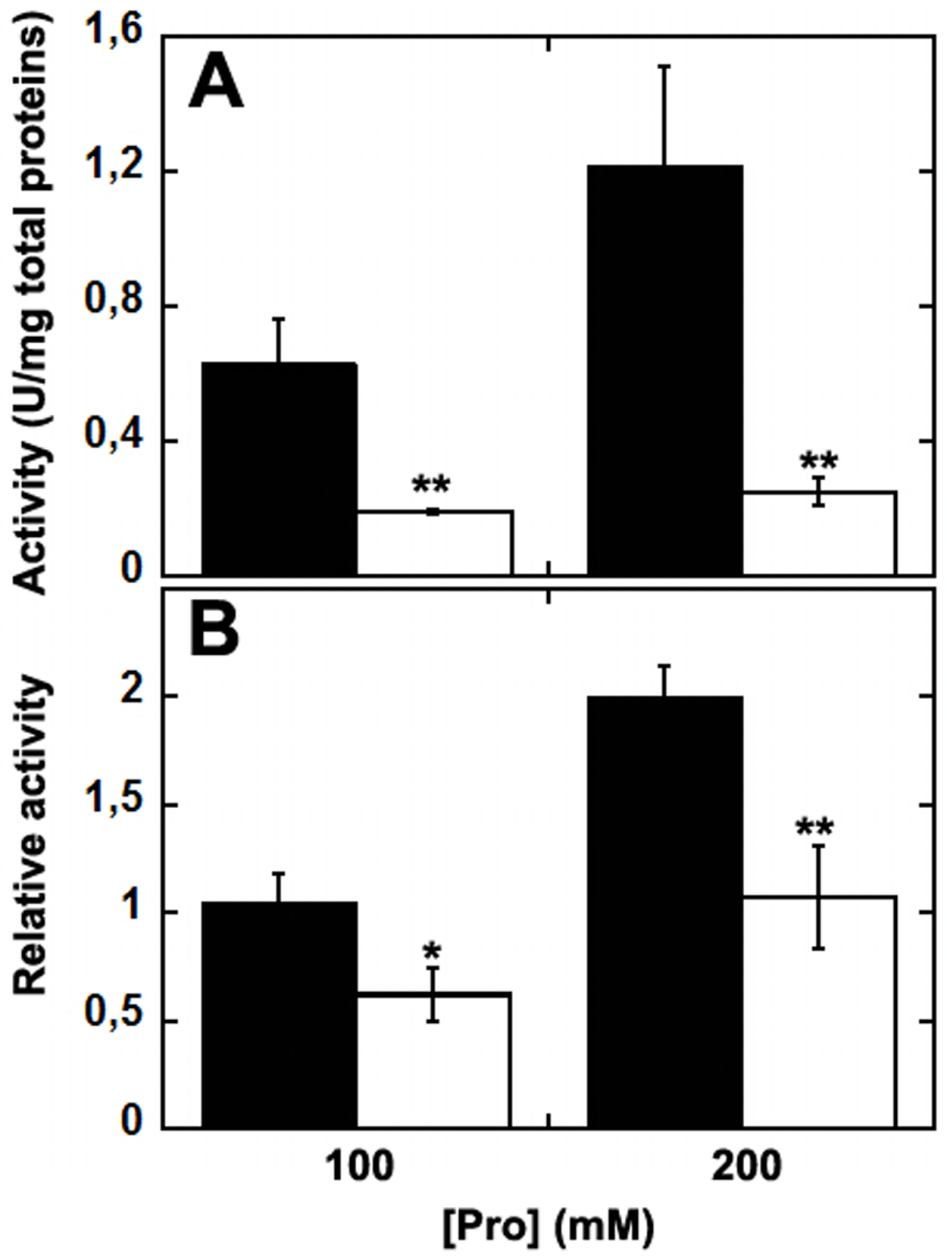
PO activity detected by the DCPIP assay method on mitochondrial fractions of U87 cells transiently transfected with wild-type or *L441P* PO and collected 48 h after transfection. Absorbance changes were corrected for the value detected in untransfected, control U87 cells. A) Activity measurements are for the entire mitochondrial protein content. B) Activity measurements were normalized to the amount of PO present in the mitochondrial fraction as assessed by Western blot analysis. The assays were performed using 100 or 200 mM L-Pro as substrate. Black bars: wild-type PO; white bars: *L441P* PO. The value is expressed as the mean ± standard error (n = 3). ** p ≤ 0.0005; * p ≤ 0.005.

To verify the effect of the *L441P* substitution on PO function, the enzymatic activity was assayed on cellular extracts of U87 PO and U87 PO *L441P* clones. When we used the o-AB assay and the previously described conditions [3], the measurements were poorly reproducible and no significant difference was observed between crude extracts from U87 cells expressing the PO variants and untransfected control cells (data not shown). For this reason, PO activity was assayed by employing the DCPIP oxidoreductase method on transfected U87 cells after isolating the mitochondrial fraction. In this case, PO activity was consistently detectable: the determined values depended on the L-Pro concentration used in the assay (Fig 4), and the activity was fully inhibited by adding 8 mM THFA (a PO inhibitor, not shown) [20], confirming the specificity of the measurements. A 3- to 5-fold higher activity was detected using mitochondrial fractions from cells expressing the wild-type protein compared to the value obtained on the same fractions from cells expressing the *L441P* PO variant (Fig 4A). When the activity values were related to the relative abundance of expressed PO (Western blot analysis showed a lower expression of the variant PO compared to the wild-type one), the relative activity of wild-type PO was approx. twofold higher than for the *L441P* variant (Fig 4B). The latter results indicate that the *L441P* substitution affects, but does not fully abolish the enzymatic activity of the flavoenzyme, while affects the protein stability.

### Effect of PO expression on L-proline, L-glutamate, and L-glutamine levels

Proline has been reported to be involved in glutamatergic transmission (see Introduction section): while proline only scarcely affects glutamate release at physiological concentration in cerebrospinal fluid, its accumulation at the synaptic terminals at a concentration above 30 μM inhibiting glutamate release [8]. To investigate whether variations in PO expression/activity might affect L-Pro, L-Glu and L-Gln cellular concentration, we determined their cellular and extracellular content in cultures of U87 cells transiently transfected with PO wild-type, PO *L441P*, and the empty pCDNA3 vector by using HPLC analysis.

**Fig 5:**
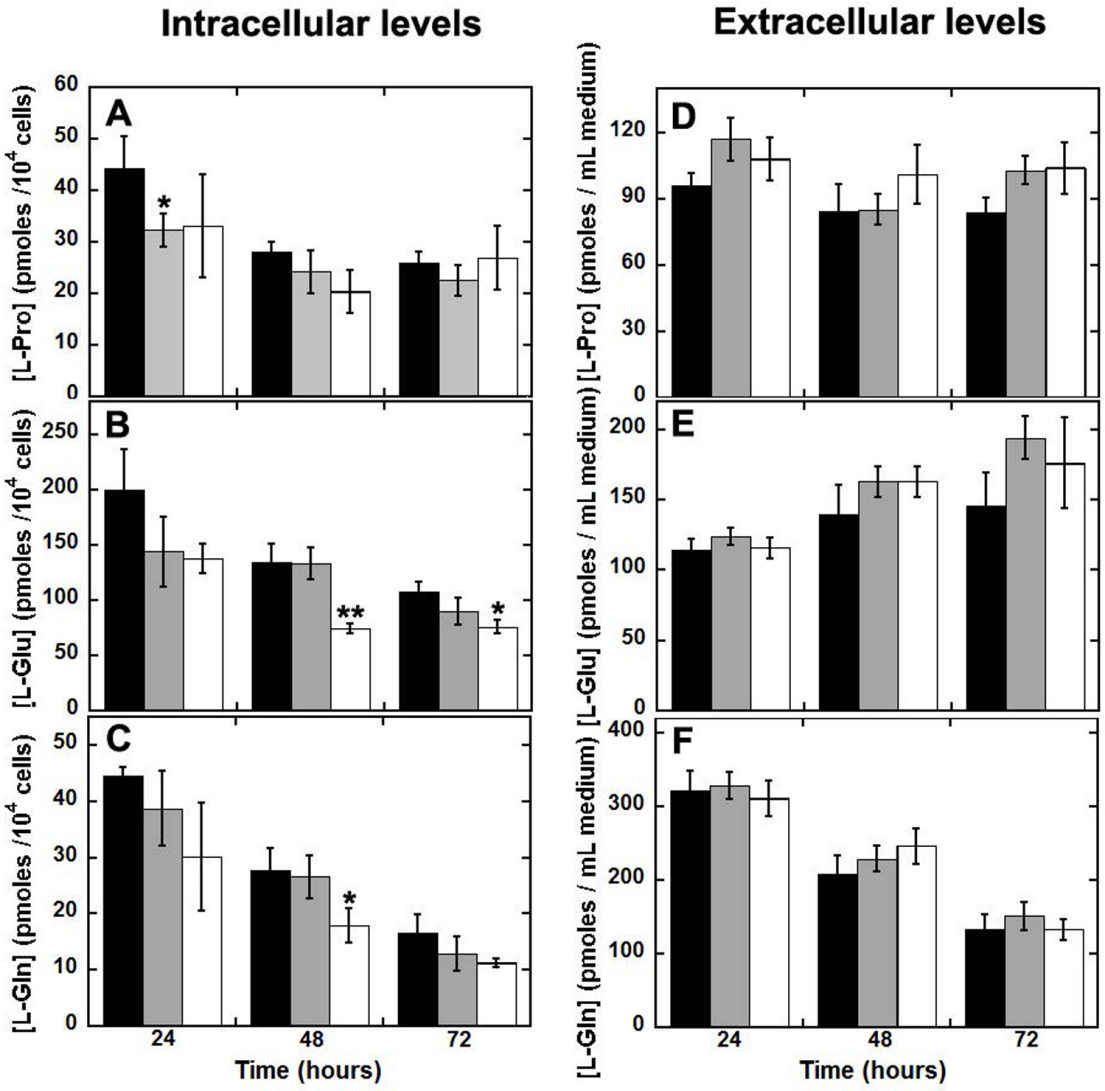
Analysis of proline, glutamate, and glutamine levels from U87 clones by HPLC. 2.7 x 10^5^ U87 control cells as well as cells transiently expressing wild-type or *L441P* PO were seeded in 6-well plates, transfected, and incubated at 37 °C, 5% CO_2_ saturation. Every 24 hours, both the culture medium and the cells were collected and analyzed for proline, glutamate, and glutamine content. Effect of the overexpression of wild-type (grey bars) or *L441P* variant (white bars) PO vs. control U87 cells transfected with the empty pCDNA3 plasmid (black bars) on the intracellular (left, panels A-C) and extracellular (right, panels D-F) levels of L-Pro, L-Glu and L-Gln. At each time point, the value is expressed as the mean ± standard error (n = 6). ** p ≤ 0.005; * p ≤ 0.05.

At 24 h after transfection, the intracellular concentration of L-Pro was lower in U87 cells expressing PO than in control cells: 32.3 ± 3.1, 33.1 ± 9.9, and 44.2 ± 6.3 pmoles/10^4^ cells for U87 cells expressing wild-type PO, *L441P* PO, and control cells, respectively, reaching statistical significance (Fig 5A, p ≤ 0.05). This result indicates that both wild-type and *L441P* variant PO are active and their expression is responsible for the increased proline intracellular consumption. This difference was less evident at longer times as a value of 22–27 pmoles/10^4^ cells was determined for all clones at 72 h (Fig 5A). Actually, when the intracellular L-Pro level is compared with the value determined at 24 h, the change at longer times between U87 cells expressing PO variants and control cells is not statistically significant (not shown).

The time course of glutamate (a proline precursor) cellular content resembled the pattern observed for L-Pro. At 24 h after transfection the lowest L-Glu levels were apparent in U87 cells overexpressing the wild-type or *L441P* PO variants, although the observed differences did not reach statistical significance (Fig 5B). For all clones, the L-Glu concentration progressively decreased with time and at 48 and 72 h it was significantly lower in cells overexpressing the *L441P* PO variant than in control cells and in cells overexpressing the wild-type PO. As compared to the value at 24 hours, the decrease in L-Glu cellular content at 48 h was stronger for the *L441P* PO clone: 50% decrease vs. 12–23% for the other clones.

Intracellular levels of L-Gln also appeared to be affected by PO overexpression: at 24 h after transfection, the most significant decrease in concentration was observed for cells expressing the *L441P* PO variant (Fig 5C). At longer times, L-Gln cellular concentration was again significantly reduced for *L441P* PO compared to wild-type expressing cells and control cells (p ≤ 0.05 at 48 h).

Notably, at 72 h after transfection the concentration of each amino acid reached a similar relative value for all three tested clones, indicating that a homeostasis was reached for the main metabolites belonging to the “proline pathway”.

Moreover, the observed decrease in amino acid levels observed at 24 h suggests that glutamate is consumed by the cells in an attempt to restore proline cellular levels depleted by the enzymatic activity of PO. In cells overexpressing the PO flavoenzyme, L-Pro is actively degraded to P5C, which can be converted into L-Glu through γ-glutamate semialdehyde (Fig 1). In turn, P5C should be also exported outside the mitochondria where P5C reductase converts it to L-Pro: in this way, L-Pro generated from L-Glu might be transferred to the mitochondrial matrix to compensate for the disequilibrium in the cycle induced by the increase in PO level and activity.

The concentrations of L-Pro, L-Glu, and L-Gln in the culture medium were also determined. Under these experimental conditions, a slightly higher extracellular L-Pro, L-Glu and L-Gln concentration was apparent for U87 cells expressing the PO variants (Fig 5D and 5F) but the change never reached statistical significance with respect to the control cells clone. For L-Gln only the extracellular concentration strongly decreased with time (Fig 5F), suggesting that it is used for cell metabolism.

### Expression of glutamate transporter

Several studies have reported impaired glutamate transporter expression levels in glioma cell lines [25]. GLAST and GLT-1 are the major Na^+^-dependent glutamate transporters in the CNS. Accordingly, we compared their levels in U87 cells stably expressing PO-EYFP or PO-EYFP *L441P* variant as well as in U87 control cells by Western blot and confocal analysis.

**Fig 6:**
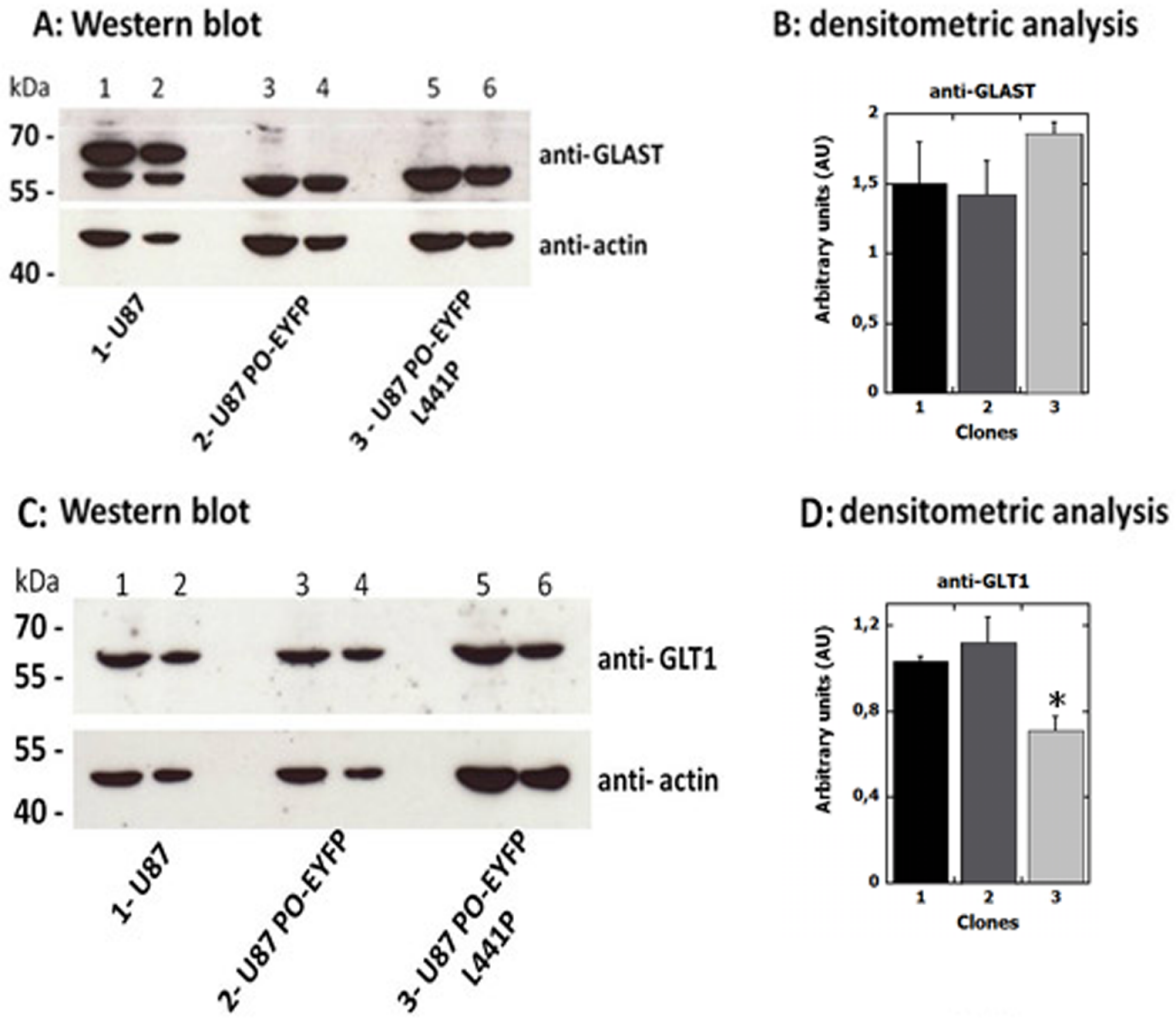
Western blot analysis of GLAST (panels A and B) and GLT-1 (panels C and D) glutamate transporters in U87 clones. The same amount of sample corresponding to 1 x 10^5^ (lanes 1, 3, 5) or 5 x 10^4^ (lanes 2, 4, 6) cells was loaded in each lane, as confirmed by detection with an anti-actin antibody as internal control. Panel A and B) Quantification analysis shows that the GLAST level (i.e., the 56-kDa band corresponding to the mature transporter) in U87 cells stably expressing PO-EYFP or PO-EYFP *L441P* is close to the level observed for U87 control cells. Panel C and D) Quantification analysis shows that control and PO-EYFP-expressing U87 cells exhibit a comparable level of expression while GLT-1 expression is 30% lower in U87 cells stably expressing PO-EYFP *L441P*. The value is expressed as the mean ± standard error (n = 3). * p ≤ 0.05.

GLAST and GLT-1 are both expressed in all the analyzed cell clones, as demonstrated by the specific immunorecognition signals detected by Western blot at the predicted molecular mass (56 kDa and 65 kDa for GLAST and GLT-1, respectively, Fig 6A and 6C). Densitometric analyses (Fig 6B) indicated that the overexpression of both the PO-EYFP variants affected GLAST cellular levels to only a minor extent: the signal corresponding to the 56-kDa immunodetected band was similar in the three U87 clones. Worthy of note is that a band in control U87 cell extracts at apparently higher mass (≈ 67 kDa), which was absent in cells expressing the PO-EYFP variants, was observed. This band might correspond to a modified form of the transporter; in particular, a glycosylated GLAST (iso)form has been reported to have the observed molecular mass [26]. Abnormal post-translational modifications, such as glycosylation, are known to affect the transporter, reducing its stability or decreasing its surface expression. Impaired glycosylation might prevent correct targeting to the plasma membrane, decreasing the glutamate reuptake capability of the cells [26–28]. GLT-1 was detected as a single band at the expected molecular mass in Western blot analysis in all clones (Fig 6C). GLT-1 expression levels in U87 control and PO-EYFP-expressing cells were similar, while a ≈ 30% lower expression level was apparent for U87 PO-EYFP *L441P* clones (Fig 6D).

**Fig 7:**
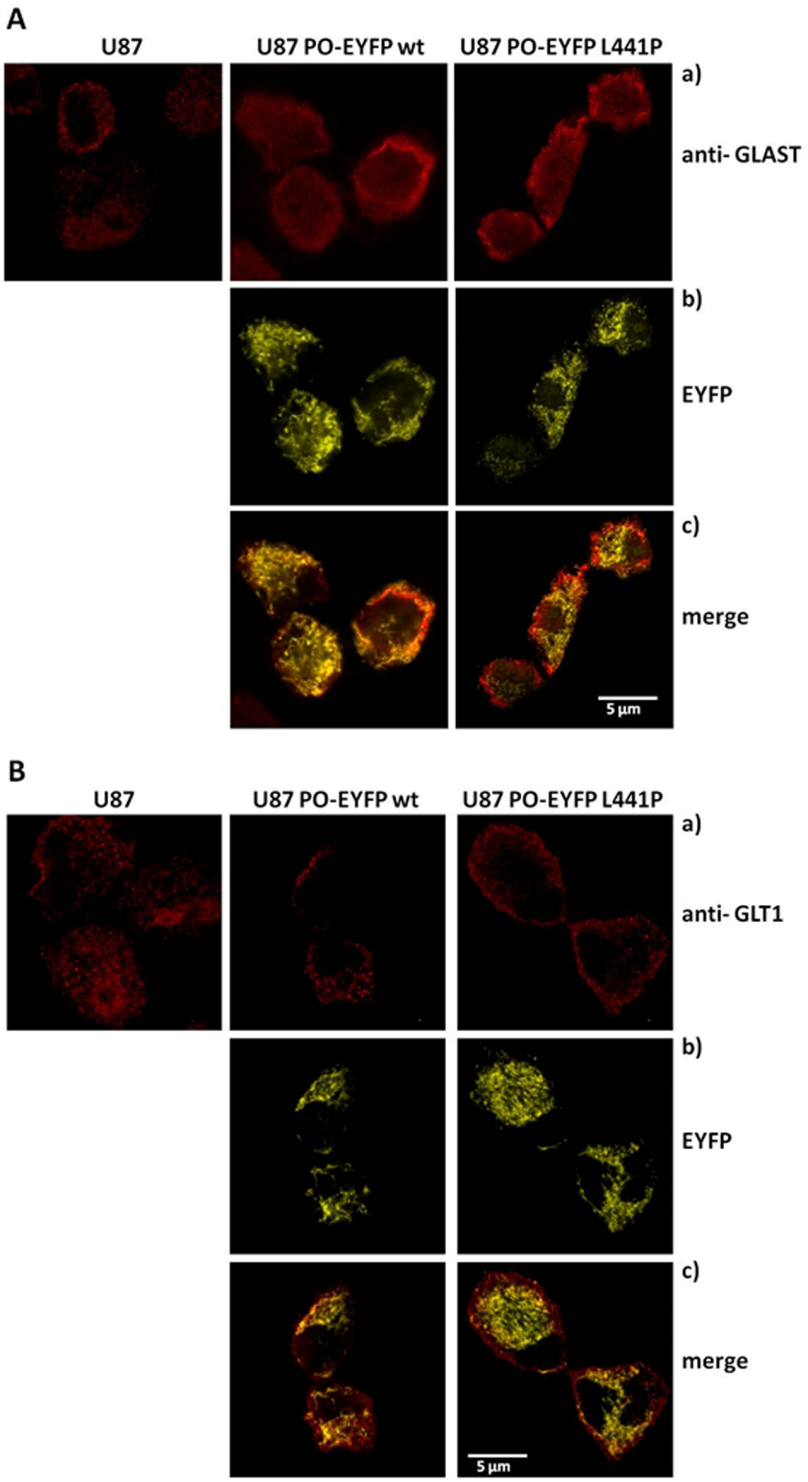
Immunohistochemical localization of GLAST and GLT-1 glutamate transporters in control U87 cells and U87 cells stably expressing PO-EYFP wild-type or *L441P*. Panel A: a) anti-GLAST staining displays predominant signal corresponding to the plasma membrane with less intense and less diffused labeling in untransfected U87 cells. b) Signals corresponding to the expressed chimeric fluorescent PO-EYFP protein (both wild-type and *L441P* variant) display a mitochondrial distribution (yellow channel). c) Overlap of the two signals corresponding to the overexpressed PO variants (yellow channel) and GLAST stain (red channel) confirm the different subcellular distribution of the glutamate transporter vs. the PO protein. Panel B): a) anti-GLT-1 staining displays a predominant signal corresponding to the plasma membrane. b) Signals corresponding to the expressed chimeric fluorescent PO protein (yellow channel) display a mitochondrial distribution. c) Overlap of the signals corresponding to the overexpressed PO variants (yellow channel) and the GLT-1 stain (red channel) confirm the different subcellular distribution of the glutamate transporter (mainly in the plasma membrane) vs. the PO proteins (in mitochondria).

Confocal analysis showed that in the cells expressing the chimeric PO variants, GLAST staining was localized at the cell membrane (as observed in control U87 cells) but it was also partially diffused in the cytosol. No significant colocalization between signals for the GLAST transporter and the chimeric PO variants was observed (Fig 7Ac). On the other hand, GLT-1 was correctly located at the cell membrane, as evident from confocal analyses (Fig 7B): the corresponding immunofluorescence signal was distributed along the cell surface where it localized in punctuate clusters (Fig 7Ba), as previously reported [29].

These results indicate that the expression levels of GLAST and GLT-1 glutamate transporters were only marginally modified by the expression of PO, which seemed to slightly alter GLAST post-translational modifications and subcellular localization/trafficking. Overall, the observed decrease in L-Pro and L-Glu levels induced by PO transient overexpression is likely not related to the levels of glutamate transporters.

## Discussion

Proline is an unusual, nonessential amino acid that may arise from dietary proteins as well as from extracellular matrix, connective tissue, and bone breakdown. As its α-nitrogen is contained within a pyrrolidine ring, L-Pro does not represent a substrate for the classical amino acid-metabolizing enzymes (decarboxylases, aminotransferases, and racemases). Worthy of note is that a family of proline metabolic enzymes evolved with specific cellular localization and regulation mechanisms [1]. L-Pro metabolism is distinct from that of the other amino acids and thus it can be reserved for special physiological functions [30]. Interestingly, L-Pro metabolic enzymes constitute a “proline pathway”, which represents a complex crossroad with other metabolic pathways (see Fig 1): this makes L-Pro a crucial amino acid in bioenergetics and regulation of cellular functions, especially in the CNS [1].

Several inborn errors of proline metabolism caused by impairments in the function of enzymes involved in the “proline pathway” have been reported. In the present study, we focused our attention on human PO, a mitochondrial flavoenzyme that catalyzes the first step of L-Pro degradation (Fig 1). The *PRODH* gene is located on chromosome 22q11, a region related to psychotic disorders. Among the reported 16 missense mutations that could affect PO function [16], the *L441P* substitution, corresponding to an allele typically found in hyperprolinemic and schizophrenic patients, was reported to result in severe reduction of PO activity [31]. Following normalization for transfection efficiency of CHO cells overexpressing PO and using a radioisotopic assay, the activity of the *L441P* PO was previously reported to be significantly lower than the wild-type (> 70% decrease) even when free FAD was added in the assay mixture [31].

In this work, the wild-type and the *L441P* PO variants were expressed in U87 glioblastoma cells as full-length or as a chimeric protein fused to EYFP at the C-terminus (to leave the N-terminal mitochondrial targeting sequence unmasked): PO expression did not alter the U87 cell growth rate (Fig 3). Confocal analysis performed on cell clones expressing PO-EYFP and PO-EYFP *L441P* showed that both enzyme variants were correctly targeted to the mitochondria (Fig 2), suggesting that the introduced substitution does not affect the protein targeting and folding.

The fused EYFP tag could affect the catalytic activity of the PO variants; therefore, the untagged wild-type and *L441P* PO variants were also expressed. By using the DCPIP assay on the purified mitochondrial fraction, we demonstrated that the activity of PO *L441P* was 3- to 5-fold lower than the wild-type enzyme (Fig 4A), a change significantly lesser than that previously reported [17, 31]. Taking into account the expression level, the specific activity of *L441P* PO was halved as compared to the wild-type enzyme (Fig 4B). Activity assay and Western blot analysis (Fig 2) indicate a negative effect of the *L441P* substitution on PO stability, as also suggested by a previous investigation [31].

The overexpression of both PO variants affects the intracellular L-Pro content: 24 h after transient transfection L-Pro content was significantly lower in U87 cells expressing wild-type or *L441P* PO than in control cells (Fig 5A). In order to expand the overview of this metabolic network we attempted to correlate PO activity with glutamate and glutamine cellular concentrations. At 24 h from transfection, the change in L-Glu levels resembled those observed for L-Pro, with lower concentrations detected in the cells clones overexpressing PO variants than in control cells (Fig 5B). In this scenario, if we consider L-Glu as a precursor of L-Pro, it is conceivable that when the proline degradation is enhanced (by the over-expression of PO variants), the “proline pathway” becomes unbalanced, thus resulting in intracellular glutamate decrease. Accordingly, even L-Gln levels were lower in U87 cells overexpressing PO variants at 24 h, although the change was not statistically significant (Fig 5C). For all three U87 cell clones differing in expression of the PO variants, the extracellular L-Pro and L-Glu content did not show a change with time. On the other hand, for L-Gln a time-dependent decrease in the culture medium was apparent for all clones (Fig 5), indicating that the observed change is mainly due to amino acid consumption during cellular metabolism. This agrees with the observed depletion of glutamine from the culture media by pancreatic ductal adenocarcinoma cells [32]. Mammalian cells have the ability to produce glutamine through GS from glutamate and ammonia in an ATP-dependent reaction. The source of free ammonia required for this reaction is still unknown [33]. A main source should be represented by asparagine, but other amino acids can also contribute, e.g. tryptophan, histidine, glycine, serine, methionine, and threonine. Notably, de novo synthesis of glutamine is not enough to accommodate all the metabolic requests, at least in tissue cultures and tumor cells.

Glioma cells surgically isolated from patients and human glioma cell lines not only show impaired uptake of glutamate but release large amounts of this amino acid, resulting in elevations of extracellular glutamate above 100 μM. Moreover, metabolic profiling of low- and high-grade glioma derived cell lines, suggests an increase in glutamate and glutamine levels during progression [34]. The observation of elevated levels of glutamate (and glycine) in tumors and peritumoral tissues compared to normal *cortex* (3.9- and 2-fold, respectively), was also confirmed in a model of rat malignant glioma [35]. In contrast with this finding, a decrease in glutamate in grade III glioma tumors or peritumoral tissues compared to normal brain tissue was also reported: 12.15 ± 3.93 μM in normal tissue vs. 6.23 ± 1.35 μM in peritumoral area and 0.80 ± 0.21 μM in tumors [36].

To better understand this pathway, and since increased L-Pro levels inhibit glutamate release [8], we investigated the expression of GLAST and GLT-1, the two major astroglial Na^+^-dependent glutamate transporters in CNS, mainly involved in maintaining a physiological, extracellular concentration of L-Glu. Glutamatergic activity is terminated through the glial uptake of the neurotransmitters: maintenance of the extracellular glutamate concentration below neurotoxic levels is a critical function of GLAST and GLT-1 [37]. In the U87 model cell system we observed that the overexpression of PO did not alter the levels of these transporters (Fig 6). Confocal analysis showed that the two transporters are mainly located in the cell membrane, but GLAST is also partially diffused in the cytosol of U87 cells expressing the PO variants (Fig 7): this result in is line with previously reported observations that these transporters are often mislocalized and their expression and translocation to the plasma membrane are reduced in glioma cells [27].

Glutamine and glutamate play pivotal roles in the malignant phenotype of brain tumors via multiple mechanisms. As glutamate is the main excitatory neurotransmitter in mammalian CNS, its extracellular concentrations are maintained in the low micromolar range. Glioma cells release large amounts of glutamate (see above), resulting in excitotoxicity through NMDA receptor activation which is believed to be the responsible for induced neuronal death in glioma surroundings [38–39], a process facilitating tumor expansion and invasion [34, 40, 41]. A growing number of studies report alterations in expression of metabotropic glutamate receptors in glioma samples. Glutamine role in tumor metabolism, including glioma, has been well documented. The growth of glia-derived tumor cells largely depends on glutamine, which is avidly taken up by cells, using mostly ASCT2 and SN1 as carriers. De Berardinis et al. showed that glioblastoma cells rely on glutamine metabolism to obtain oxaloacetate for Krebs cycle and NADPH for biosynthetic pathways, thus supporting cell growth and proliferation [42]. Finally, although alterations in proline levels have been described in several types of tumors, specific information is lacking for glioma. However, Panosyan et al. reported that several amino acid metabolizing enzymes are altered in glioma: decreased PO levels compared to normal tissue is a common feature of all glioma subtypes [43].

Taken together, our results demonstrate the physiological relevance of PO activity in the intriguing scenario that links the “proline pathway” with other fundamental metabolic pathways and neuroactive amino acids. Clearly, PO activity also affects L-Glu and L-Gln intracellular levels by altering L-Pro levels. Further investigations are required to elucidate the mechanism by which L-Pro might exert its function as a “putative neuromodulator” under physiological and pathological conditions. Understanding the complex biochemical network linking proline to glutamate in the CNS will pave the way to the design of new drugs targeting the enzymes modulating the concentration of these amino acids as a new therapeutic target for schizophrenia.

## Supporting information

S1 Fig Analysis of glutamine levels in U87 cells transfected with pCDNA3 by HPLC. A) The cells were detached by trypsin treatment (black line) or by scraping (dotted line) in ice cold PBS, and collected in aliquots. Cell extracts were prepared following the procedure detailed in the Materials and methods section. The detected glutamine content is significantly lower in the scraped cells. B) A fixed amount of glutamine was added as internal standard to a sample of trypsin-treated cells upon resuspension in ice cold 5% TCA. The corresponding chromatogram (dotted line) is compared to the one for the sample without addition (black line). The analysis indicated a very high of added glutamine following the extraction procedure, thus demonstrating the accuracy of the determination of its level in the different samples.
(TIF)

S1 Table HPLC analysis of glutamine levels in U87 pCDNA3 transfected cells, collected by trypsin treatment (Tryp) or by scraping (Scrap). (PDF)

S1 File Original unadjusted blots of Figs 2 and 6. (PDF)

## Abbreviations

CNS: central nervous system
DCPIP: dichlorophenolindophenol
EAAT: excitatory amino acid transporter
EYFP: enhanced yellow fluorescent protein
FAD: flavin adenine dinucleotide
FBS: fetal bovine serum
GLS: glutaminase
GS: glutammine synthase
HPI: hyperprolinemia type I
MTT: (3-[4,5-dimethylthiazol-2-yl]-2,5-diphenyltetrazolium bromide
NMDA: N-methyl-D-aspartate
*o*-AB: *o*-aminobenzaldehyde
OAT: ornithine aminotransferase
P5C: Δ^1^-pyrroline-5-carboxylic acid
PBS: phosphate buffer saline
PC5DH: Δ^1^-pyrroline-5-carboxylate dehydrogenase
PC5R: pyrroline-5-carboxylate reductase 1
PO: proline oxidase
PRODH: proline dehydrogenase
TBS: Tris-buffered saline
TPBS: phosphate buffer saline added with 0.1% Tween-20
TTBS: Tris-buffered saline added with 0.1% Tween-20

## References

[1] Phang JM, Hu CA, Valle D. Disorders of proline and hydroxyproline metabolism The metabolic and molecular bases of inherited disease, 8th ed Scriver C.R., Beaudet A.R., Sly W., Valle D (eds.), McGraw-Hill, 2001; 1821–1838.

[2] Maxwell SA, Rivera A. Proline oxidase induces apoptosis in tumor cells, and its expression is frequently absent or reduced in renal carcinomas. J Biol Chem. 2003; 278(11):9784–9789. doi: 10.1074/jbc.M210012200 12514185 10.1074/jbc.M210012200

[3] Pandhare J, Donalds SP, Cooper SK, Phang JM. Regulation and function of proline oxidase under nutrient stress. J Cell Biochem. 2009; 107(4):759–768. doi: 10.1002/jcb.22174 19415679 10.1002/jcb.22174 PMC2801574

[4] Servet C, Ghelis T, Richard L, Zilberstein A, Savoure A. Proline dehydrogenase: a key enzyme controlling cellular homeostasis. Front Biosci. 2012; 17(1):607–620. 10.2741/3947 22201764

[5] Krishnan N, Dickman MB, Becker DF. Proline modulates the intracellular redox environment and protects mammalian cells against oxidative stress. Free Radic Biol Med. 2008; 44(4):671–681. doi: 10.1016/j.freeradbiomed.2007.10.054 18036351 10.1016/j.freeradbiomed.2007.10.054 PMC2268104

[6] Tanner JJ. Structural biology of proline catabolism. Amino Acids. 2008; 35(4):719–730. doi: 10.1007/s00726-008-0062-5 18369526 10.1007/s00726-008-0062-5 PMC2664619

[7] Phang JM, Liu W. Proline metabolism and cancer. Front Biosci. 2012; 17(1):1835–1845. 10.2741/4022 PMC7467630 22201839

[8] Cohen SM, Nadler JV. Proline induced inhibition of glutamate release in hippocampal area CA1. Brain Res. 1997; 769(2):333–339. 9374203 10.1016/s0006-8993(97)00721-x

[9] Wyse AT, Netto CA. Behavioral and neurochemical effects of proline. Metab Brain Dis. 2011; 26(3):159–172. doi: 10.1007/s11011-011-9246-x 21643764 10.1007/s11011-011-9246-x

[10] Yu XC, Zhang W, Oldham A, Buxton E, Patel S, Nghi N, et al Discovery and characterization of potent small molecule inhibitors of the high-affinity proline transporter. Neurosci Lett. 2009; 451(3):212–216. doi: 10.1016/j.neulet.2009.01.018 19159658 10.1016/j.neulet.2009.01.018

[11] Fremau RT Jr, Caron MG, Blakely RD. Molecular cloning and expression of high affinity proline transporter expressed in putative glutamatergic pathway of rat brain. Neuron. 1992; 8(5):915–926. 1350201 10.1016/0896-6273(92)90206-s

[12] Renick SE, Kleven DT, Chan J, Stenius K, Milner TA, Pickel VM, et al The mammalian brain high-affinity L-proline transporter is enriched preferentially in synaptic vescicles in a subpopulation of excitatory nerve terminals rat forebrain. J Neurosci. 1999; 19(1):21–33. 9870934 10.1523/JNEUROSCI.19-01-00021.1999 PMC6782366

[13] Jacquet H, Raux G, Thibaut F, Hecketsweiler B, Houy E, Demilly C, et al PRODH mutation and hyperprolinemia in a subset of schizophrenic patients. Hum Mol Genet. 2002; 11(19):2243–2249. 12217952 10.1093/hmg/11.19.2243

[14] Liu H, Heath SC, Sobin C, Roos JL, Galke BL, Blundell ML, et al Genetic variation at the 22q11 PRODH2/DGCR6 locus presents an unusual pattern and increases susceptibility to schizophrenia. Proc Natl Acad Sci USA. 2002; 99(6):3717–3722. doi: 10.1073/pnas.042700699 11891283 10.1073/pnas.042700699 PMC122590

[15] Williams HJ, Williams N, Spurlock G, Norton N, Zammit S, Kirov G, et al Detailed analysis of PRODH and PsPRODH reveals no association with schizophrenia. Am J Med Genet B Neuropsychiatr Genet. 2003; 120B(1):42–46. doi: 10.1002/ajmg.b.20049 12815738 10.1002/ajmg.b.20049

[16] Mitsubuchi H, Nakamura K, Matzumoto S, Endo F. Inborn errors of proline metabolism. J Nutr. 2008; 138(10):2016–2020. 10.1093/jn/138.10.2016S 18806117

[17] Bender HU, Almashanu S, Steel G, Hu CA, Lin WW, Willis A, et al Functional consequences of PRODH missense mutations. Am J Hum Genet. 2005; 76(3):409–420. doi: 10.1086/428142 15662599 10.1086/428142 PMC1196393

[18] Clelland CL, Read LL, Baraldi AN, Bart CP, Pappas CA, Panek LJ, et al Evidence for association of hyperprolinemia with schizophrenia and a measure of clinical outcome. Schizophr Res. 2011; 131(1–3):139–145. doi: 10.1016/j.schres.2011.05.006 21645996 10.1016/j.schres.2011.05.006 PMC3161723

[19] Orešič M, Tang J, Seppänen-Laasko T, Mattila I, Saarni SE, Saarni SI, et al Metabolome in schizophrenia and other psychotic disorders: a general population–based study. Genome Med. 2011; 3(3):19–33. doi: 10.1186/gm233 21429189 10.1186/gm233 PMC3092104

[20] Tallarita E, Pollegioni L, Servi S, Molla G. Expression in *Escherichia coli* of the catalytic domain of human proline oxidase. Protein Expr Purif. 2012; 82(2):345–351. doi: 10.1016/j.pep.2012.01.021 22333530 10.1016/j.pep.2012.01.021

[21] Sacchi S, Bernasconi M, Martineau M, Mothet JP, Ruzzene M, Pilone MS, et al pLG72 Modulates Intracellular D-serine levels through its interaction with D-amino acid oxidase—effects on schizophrenia susceptibility. J Biol Chem. 2008; 283(32):22244–22256. doi: 10.1074/jbc.M709153200 18544534 10.1074/jbc.M709153200

[22] Sacchi S, Cappelletti P, Giovannardi S, Pollegioni L. Evidence of the interaction of D-amino acid oxidase with pLG72 in glial cell line. Mol Cell Neurosci. 2011; 48(1):20–28. doi: 10.1016/j.mcn.2011.06.001 21679769 10.1016/j.mcn.2011.06.001

[23] Shoji K, Mariotto S, Ciampa AR, Suzuki H. Regulation of serine racemase activity by D-serine and nitric oxide in human glioblastoma cells. Neurosci Lett. 2006; 392(1–2):75–78. doi: 10.1016/j.neulet.2005.08.063 16182447 10.1016/j.neulet.2005.08.063

[24] Maynard TM, Meechan DW, Dudevoir ML, Gopalakrishna D, Peters AZ, Heindel CC, et al Mitochondrial localization and function of a subset of 22q11 deletion syndrome candidate genes. Mol Cell Neurosci. 2008; 39(3):439–451. doi: 10.1016/j.mcn.2008.07.027 18775783 10.1016/j.mcn.2008.07.027 PMC2729512

[25] Maragakis NJ, Rothstein JD. Glutamate transporters: animal models to neurologic disease. Neurobiol Dis. 2004; 15(3):461–473. doi: 10.1016/j.nbd.2003.12.007 15056453 10.1016/j.nbd.2003.12.007

[26] Escartin C, Brouillet E, Gubellini P, Trioulier Y, Jacquard C, Smadja C, et al Ciliary neurotrophic factor activates astrocytes, redistributes their glutamate transporters GLAST and GLT-1 to raft microdomains, and improves glutamate handling *in vivo*. J Neurosci. 2006; 26(22):5978–5989. doi: 10.1523/JNEUROSCI.0302-06.2006 16738240 10.1523/JNEUROSCI.0302-06.2006 PMC6675222

[27] Ye ZC, Rothstein JD, Sontheimer H. Compromised glutamate transport in human glioma cells: reduction-mislocalization of sodium-dependent glutamate transporters and enhanced activity of cystine-glutamate exchange. J Neurosci. 1999; 19(24):10767–10777. 10594060 10.1523/JNEUROSCI.19-24-10767.1999 PMC6784962

[28] Bauer D, Haroutunian V, Meador-Woodruff JH, McCullumsmith RE. Abnormal glycosylation of EAAT1 and EAAT2 in prefrontal cortex of elderly patients with schizophrenia. Schizophr Res. 2010; 117(1):1–14. 19716271 10.1016/j.schres.2009.07.025 PMC2822023

[29] Lee A, Anderson AR, Beasley SJ, Barnett NL, Poronnik P, Powa DV. A new splice variant of the glutamate–aspartate transporter: cloning and immunolocalization of GLAST1c in rat, pig and human brains. J Chem Neuroanat. 2012; 43(1):52–63. doi: 10.1016/j.jchemneu.2011.10.005 22026960 10.1016/j.jchemneu.2011.10.005

[30] Phang JM, Donald SP, Pandhare J, Liu Y. The metabolism of proline, a stress substrate, modulates carcinogenic pathways. Amino Acids. 2008; 35(4):681–690. doi: 10.1007/s00726-008-0063-4 18401543 10.1007/s00726-008-0063-4

[31] Willis A, Bender HU, Steel G, Valle D. *Prodh* variants and risk for schizophrenia. Amino Acids. 2008; 35(4):673–679. doi: 10.1007/s00726-008-0111-0 18528746 10.1007/s00726-008-0111-0

[32] Olivares O, Mayers JR, Gouirand V, Torrence M, Gicquel T, Borge L, et al Collagen-derived proline promotes pancreatic ductal adenocarcinoma cell survival under nutrient limited conditions. Nat Commun. 2017; 8:16031 doi: 10.1038/ncomms16031 28685754 10.1038/ncomms16031 PMC5504351

[33] Zhang J, Pavlova NN, Thompson CB. Cancer cell metabolism: the essential role of the nonessential amino acid, glutamine. EMBO J. 2017; 36(10):1302–1315. doi: 10.15252/embj.201696151 28420743 10.15252/embj.201696151 PMC5430235

[34] Behrens PF, Langemann H, Strohschein R, Draeger J, Hennig J. Extracellular glutamate and other metabolites in and around RG2 rat glioma: an intracerebral microdialysis study. J Neurooncol. 2000; 47(1):11–22. 10930095 10.1023/a:1006426917654

[35] Bianchi L, De Micheli E, Bricolo A, Ballini C, Fattori M, Venturi C, et al Extracellular levels of amino acids and choline in human high grade gliomas: an intraoperative microdialysis study. Neurochem Res. 2004; 29(1):325–34. 14992293 10.1023/b:nere.0000010462.72557.6d

[36] Shao W, Gu J, Huang C, Liu D, Huang H, Huang Z, et al Malignancy-associated metabolic profiling of human glioma cell lines using 1H NMR spectroscopy. Mol Cancer. 2014; 13:197 doi: 10.1186/1476-4598-13-197 25163530 10.1186/1476-4598-13-197 PMC4158044

[37] Lopez-Bayghen E, Ortega A. Glial glutamate transporters: new actors in brain signaling. IUBMB Life. 2011; 63(10):816–823. doi: 10.1002/iub.536 21901813 10.1002/iub.536

[38] Ye ZC, Sontheimer H. Glioma cells release excitotoxic concentrations of glutamate. Cancer Res. 1999; 59(17):4383–4391. 10485487

[39] Olney JW. Excitotoxicity, apoptosis and neuropsychiatric disorders. Curr Opin Pharmacol. 2003; 3(1):101–9. 12550750

[40] Pereira MSL, Klamt F, Thomé CC, Worm PV, de Oliveira DL. Metabotropic glutamate receptors as a new therapeutic target for malignant gliomas. Oncotarget. 2017; 8(13):22279–22298. doi: 10.18632/oncotarget.15299 28212543 10.18632/oncotarget.15299 PMC5400663

[41] Takano T, Lin JH, Arcuino G, Gao Q, Yang J, Nedergaard M. Glutamate release promotes growth of malignant gliomas. Nat Med. 2001; 7(9):1010–1015. doi: 10.1038/nm0901-1010 11533703 10.1038/nm0901-1010

[42] DeBerardinis RJ, Mancuso A, Daikhin E, Nissim I, Yudkoff M, Wehrli S, et al Beyond aerobic glycolysis: transformed cells can engage in glutamine metabolism that exceeds the requirement for protein and nucleotide synthesis. Proc Natl Acad Sci U S A. 2007; 104(49):19345–50. doi: 10.1073/pnas.0709747104 18032601 10.1073/pnas.0709747104 PMC2148292

[43] Panosyan EH, Lin HJ, Koster J, Lasky JL 3rd. In search of druggable targets for GBM amino acid metabolism. BMC Cancer. 2017; 17(1):162 doi: 10.1186/s12885-017-3148-1 28245795 10.1186/s12885-017-3148-1 PMC5331648

