# Supplementary figures and images for: Proline oxidase controls proline, glutamate, and glutamine cellular concentrations in a U87 glioblastoma cell line
Source: PLoS One. 2018 Apr 25;13(4):e0196283. doi: 10.1371/journal.pone.0196283 (PMC5918996; doi:10.1371/journal.pone.0196283)

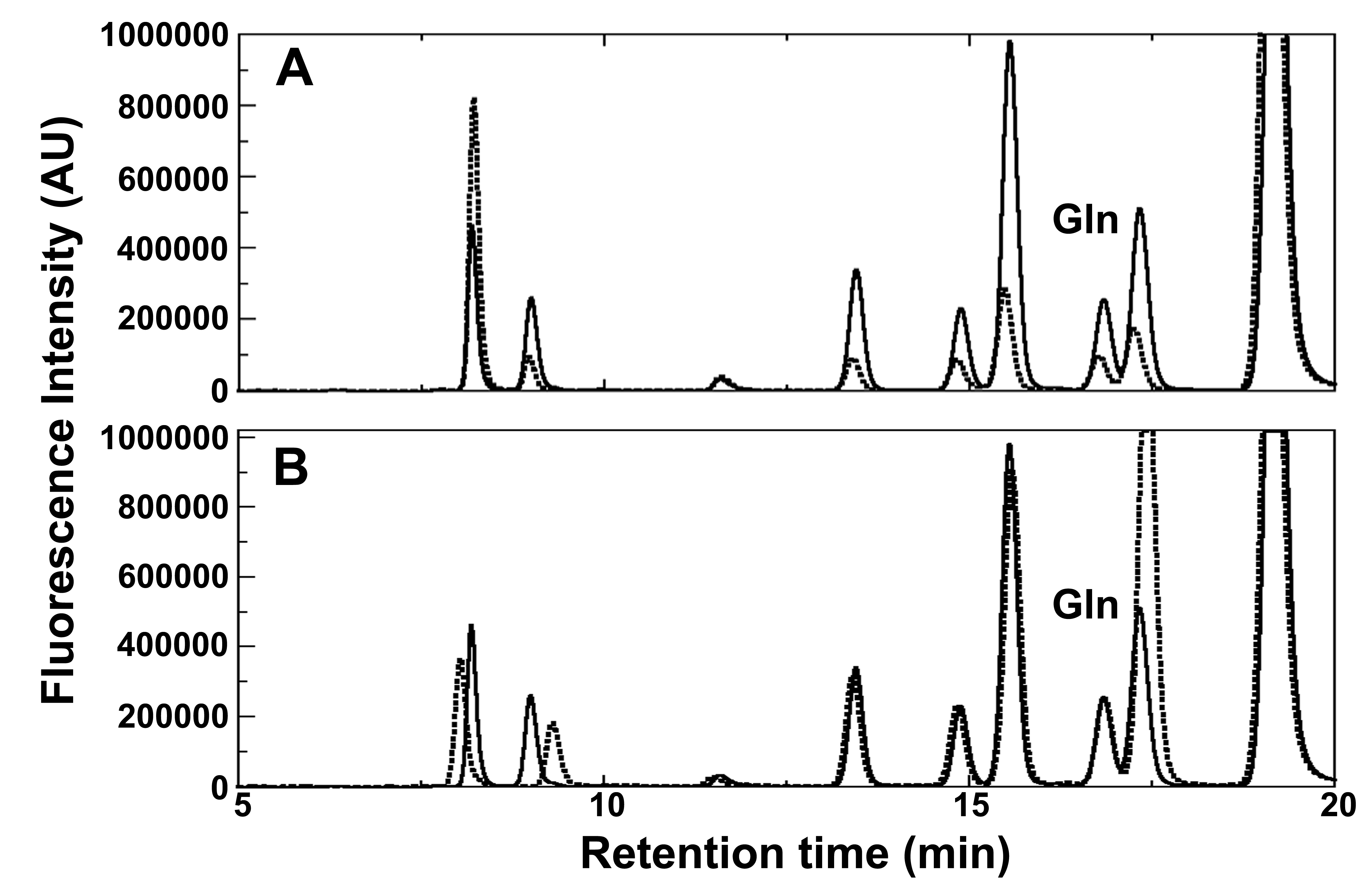

Supplement: S1 Fig — A) The cells were detached by trypsin treatment (black line) or by scraping (dotted line) in ice cold PBS, and collected in aliquots. Cell extracts were prepared following the procedure detailed in the Materials and methods section. The detected glutamine content is significantly lower in the scraped cells. B) A fixed amount of glutamine was added as internal standard to a sample of trypsin-treated cells upon resuspension in ice cold 5% TCA. The corresponding chromatogram (dotted line) is compared to the one for the sample without addition (black line). The analysis indicated a very high of added glutamine following the extraction procedure, thus demonstrating the accuracy of the determination of its level in the different samples. (TIF) [file pone.0196283.s001.tif]
