# Supplementary material for: Proline oxidase controls proline, glutamate, and glutamine cellular concentrations in a U87 glioblastoma cell line
Source: PLoS One. 2018 Apr 25;13(4):e0196283. doi: 10.1371/journal.pone.0196283 (PMC5918996; doi:10.1371/journal.pone.0196283)
Supplement: S1 Table — (PDF) [file pone.0196283.s002.pdf]

Cells	pmoles of Gln (injected sample)	Gln Peak Area	Expected Peak Area	Gln recovery (%)
Tryp	198*	8567063		
Scrap	66.5*	2819690		
Tryp + Gln	198*+180**	17928992	16346810	109
Scrap + Gln	66.5*+50**	4563432	4917797	93

*measured amount of glutamine in the injected sample; ** amount of glutamine added to the samples as internal standard.
