# Supplementary material for: Proline oxidase controls proline, glutamate, and glutamine cellular concentrations in a U87 glioblastoma cell line
Source: PLoS One. 2018 Apr 25;13(4):e0196283. doi: 10.1371/journal.pone.0196283 (PMC5918996; doi:10.1371/journal.pone.0196283)
Supplement: S1 File — (PDF) [file pone.0196283.s003.pdf]

Original unadjusted blots

Fig 2A: Western Blot analysis repeated in response to reviewer 1:

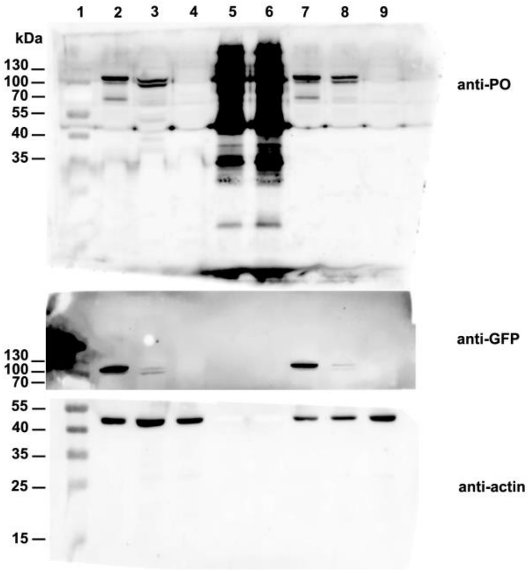

Fig 2A. Expression of PO in stably transfected U87 clones. Western blot analysis performed using mouse anti-GFP or rabbit anti-PO antibodies confirms the expression of both the fluorescent PO-EYFP wild-type and *L441P* fusion proteins in transfected U87 cells. The same amount of sample, corresponding to 5 x 10^4 cells was loaded in each lane. Lane 1= MW; lane 2= U87-PO-EYFP wt; lane 3= U87-PO-EYFP L441P; lane 4= U87; lane 5= 50 ng PO-BarrelN-His; lane 6 = 200 ng PO-BarrelN-His; lane 7 = U87-PO-EYFP wt; lane 8= U87-PO-EYFP L441P; lane 9= U87

Fig 2B

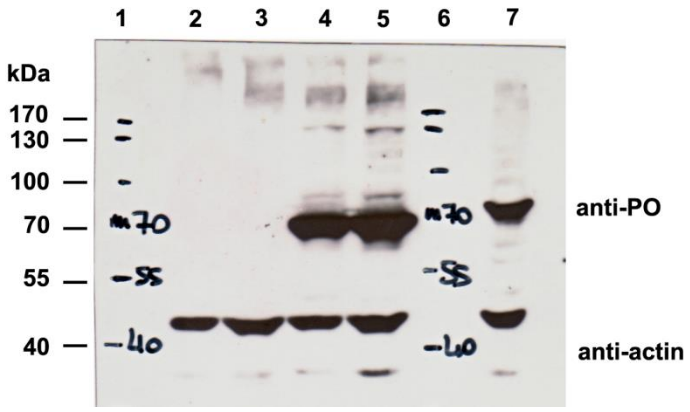

Fig 2B. Expression of PO in stably transfected U87 clones. Western blot analysis performed using rabbit anti-PO antibodies confirms the expression of both the untagged proteins. Lane 1 and 6= MW; lane 2= 50 µg of crude extract of U87; lane 3 = 60 µg of crude extract of U87; lane 4= 50 µg of crude extract of U87 pCDNA3 PO wt (reported in the manuscript's figure); lane 5= 60 µg of crude extract of U87 pCDNA3 PO wt; lane 7= 50 µg of crude extract of U87 pCDNA3 PO L441P (reported in the manuscript's figure)

Fig 6A:

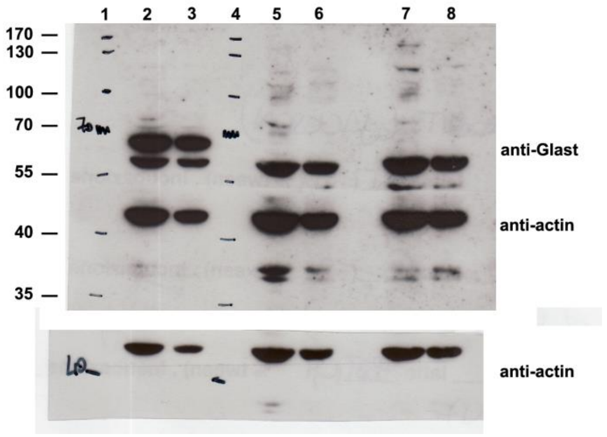

Fig 6A. Western blot analysis of GLAST glutamate transporter in U87 clones. The same amount of sample corresponding to 1 x 10^5 (lanes 2, 5, 7) or 5 x 10^4 (lanes 3, 6, 8) cells was loaded in each lane, as confirmed by detection with an anti-actin antibody as internal control (The anti-actin analysis reported in the manuscript is the second below. Lane 1 and 4 = MW; lane 2 and 3 = U87; lane 5 and 6 = U87 PO-EYFP; lane 7 and 8 = U87 PO-EYFP L441P

Fig 6C

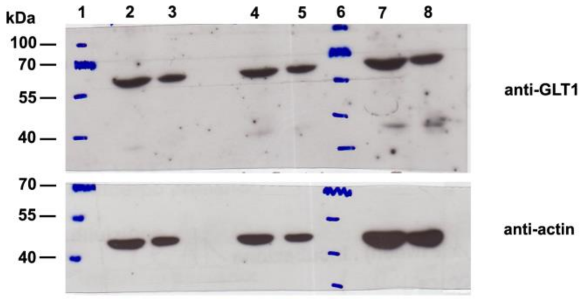

Fig 6. Western blot analysis of GLT-1 glutamate transporter in U87 clones. The same amount of sample corresponding to 1 x 10^5 (lanes 2, 4, 7) or 5 x 10^4 (lanes 3, 5, 8) cells was loaded in each lane, as confirmed by detection with an anti-actin antibody as internal control. Lane 1 and 6 = MW; lane 2 and 3 = U87; lane 4 and 5 = U87 PO-EYFP; lane 7 and 8 = U87 PO-EYFP L441P
